# Carbolic-Acid Poisoning: An Experimental Investigation

**Published:** 1916-03-25

**Authors:** 


### CARBOLIC-ACID POISONING.

### An Experimental Investigation.

POISONING by carbolic acid is hardly among the rare experiences of general practice. The acid is popular among those bent upon self-destruction, and, as it is used for purposes of domestic sanitation, cases of its accidental administration occur every now and again. In view of these not very infrequent experiences it is somewhat curious that a measure of uncertainty exists in reference to the best method of treatment to adopt. Emetics may fail to act, presumably as a result of the anæsthetic action of the poison on the gastric mucous membrane. Apomorphine injected subcutaneously may be more successful, but in the main reliance has to be placed on washing out the stomach. The question of uncertainty appears when the fluid to be used for this purpose has to be decided. Is plain water the best, or should some medicinal agent be added? Among such agents sodium sulphate, magnesium sulphate, and alcohol are perhaps most frequently mentioned, though other substances—*e.g.*, tincture of iodine, albumen, and permanganate of potassium—have also been proposed. The somewhat contradictory opinions which have been expressed on the subject have induced Dr. David L. Macht * to undertake an experimental investigation with a view to settle the question. His observations and opinions are of great importance.

First, on the question of alcohol he comes to a confident decision that the use of this agent in carbolic-acid poisoning is not only not helpful, but is as a matter of fact prejudicial, for it actually promotes the absorption of the poison. The experiments offer an interesting suggestion as to the manner in which the suggestion of the use of alcohol arose. They show that very different results follow according as the alcohol is given before or after the administration of carbolic acid. In the former case the animal is able better to withstand the effects of phenol, as if the affinity of the nerve cells for alcohol led to a temporary union which opposed the attack of the acid. On the other hand, when alcohol was given simultaneously with or immediately after the phenol, the animal succumbed more readily than when phenol was given alone. These results may help to show how the recommendation of alcohol arose, for in a certain number of cases of poisoning by phenol alcohol in considerable quantities has been taken in advance, and the recovery of such patient would easily give rise to a suggestion of the antidotal value of the spirit. Further, it is more or less widely known that in carbolic-acid "burns" of the skin alcohol is an excellent application, thus affording a second basis for the practice here discussed. In this connection it is noteworthy that of ten cases of carbolic-acid poisoning in which the persons drank the poison in various alcoholic drinks, such as schnapps, brandy, rum, or beer, no fewer than nine succumbed. In regard to lavage with plain water, the experiments seem to show pretty conclusively that this has a quite decided value, and this is the more important seeing that the practice has been challenged. If commenced early a good effect is promoted, and in individual cases where there has been delay even of an hour or more recovery has followed.

* *Johns Hopkins Hospital Bulletin*, April 1915.

### THE VALUE OF SULPHATES.

The principal positive interest of Dr. Macht's investigation relates to the use of sodium sulphate, which in strong solutions gave decidedly better results than when water alone was employed. How exactly the sulphate acts is a matter of uncertainty. By some it is stated that harmless sulpho-carbolates are formed, but the probability is that the action is not a chemical one. The salt appears to hinder the absorption of the poison, and by purgation it helps to remove it from the alimentary canal. Magnesium sulphate in these two respects may claim equal value, but it has one disadvantage—namely, that in so far as it is absorbed it has a depressing action, and might therefore add to the deleterious effects of the carbolic acid. Epsom salts have so wide a popularity that it is not always remembered that they have produced poisonous results; there are at least five or six fatal cases on record. Hence it would be well in a case of carbolic-acid poisoning to prefer the sodium salt, though in an emergency the magnesium sulphate might have to be accepted, taking care in such circumstances to leave as little as possible in the stomach.

Two other points may be noted. On the question of the minimum fatal dose of carbolic acid Dr. Macht quotes an instance of a girl of twenty-four years in whom 23 grains of phenol produced death, and another in which a drachm caused a fatal result in a girl of seventeen years. Usually much larger quantities are taken with suicidal intent—roughly, about an ounce, and not always with a fatal issue. The presence of food in the stomach is a favourable condition, for it combines with the acid and may thus postpone and hinder absorption. Hence a given dose will be less active, and the delay offers a greater opportunity for effective treatment. A second practical point brings us back to the use of alcohol. Accepting the view that to use this agent as an antidote and to wash out the stomach is a mistaken practice, it yet remains true that alcohol has its *rôle* in carbolic-acid poisoning in man. The feeble pulse and other signs of collapse obviously claim stimulating agencies. These symptoms may be less marked in cats and dogs, but they are prominent features in the human subject. To meet them such measures as warmth to the surface and the hypodermic use of strychnine are suggested. In addition there is a manifest claim for the stimulating action of alcohol or one of its allies. Brandy may thus be given *per rectum*, or ether may be injected under the skin.

